# WE♡CARE: A CURRICULUM FOR CAREGIVERS WITH DEVELOPMENTAL DISABILITIES

**DOI:** 10.1093/geroni/igae098.2686

**Published:** 2024-12-31

**Authors:** Britney Burkart

**Affiliations:** University of Missouri-Kansas City’s Institute for Human Development: Health and Aging, Kansas City, Missouri, United States

## Abstract

Many adults with developmental disabilities (DD) live at home with their caregivers. However, there are limited resources available to individuals with DD living in mutual caregiving arrangements, a scenario in which adults with DD become more responsible for providing care to an aging loved one. This poster presentation will provide an opportunity to learn more about WE♡CARE, a curriculum developed for adults with DD living in a mutual caregiving arrangement. A mixed methods approach was employed to measure curriculum impact and identify lessons learned surrounding curriculum implementation. Observational data collected during the initial pilot program indicated that adults with DD can benefit from facilitated programs such as WE♡CARE. For example, participants of the pilot program not only learned to recognize when their loved one might need support, but also to develop confidence as a caregiver. Quantitative data measuring change in knowledge among participants showed improvement from pre-session to post-session. A confidence assessment administered before and after participation did show a statistically significant increase in confidence from pre to post (p < 0.05). While the curriculum offers great value to individuals with DD and their loved ones, the pilot program shed light on the need to modify protocols, considering physical and cognitive accessibility and adaptability of facilitators to individual needs. As the population ages, mutual caregiving relationships will become more common, and the knowledge and skill sets embedded in this curriculum can support the growing number of mutual caregivers throughout the country.

